# Radiographic Analysis of Lower Limb Alignment in Patients with Knee Osteoarthritis

**DOI:** 10.3390/tomography12070103

**Published:** 2026-07-07

**Authors:** Ozden Bedre Duygu, Figen Govsa, Anil Murat Ozturk, Mehmet Alp Ozmen

**Affiliations:** 1 Department of Anatomy, Faculty of Medicine, Izmir Bakircay University, 35660 Izmir, Turkey; 2 Department of Anatomy, Faculty of Medicine, Ege University, 35040 Izmir, Turkey; 3 Department of Orthopedics and Traumatology, Faculty of Medicine, Ege University, 35040 Izmir, Turkey; amuratozturk@yahoo.com; 4 Department of Orthopedics and Traumatology, Izmir City Hospital, 35540 Izmir, Turkey; mehmetalpozmen@gmail.com

**Keywords:** knee osteoarthritis, lower limb alignment, radiography

## Abstract

*Background and Objectives:* Knee osteoarthritis (OA) is a prevalent musculoskeletal disorder. This study aims to assess the hip-knee-ankle anatomical alignment in patients with OA at different stages. *Materials and Methods:* Radiological images of 200 OA patients were analyzed to measure parameters such as femoral and tibial lengths (both anatomical and mechanical), abductor length, hip center length, femoral offset, collum femoris length, and shaft angle using ImageJ software (version 1.53k; National Institutes of Health, Bethesda, MD, USA). *Results:* The female-to-male ratio was 2.17:1. Male participants exhibited greater values for femoral and tibial lengths, abductor length, hip center length, femoral offset, collum femoris length, and collum femoris shaft angle, whereas females showed higher Q angle, hip–knee–ankle angle, mechanical lateral distal femoral angle, mechanical medial proximal tibial angle, and femoral angle measurements. Significant differences in both linear and angular parameters were observed among age groups. Q angle, hip–knee–ankle angle, femoral mechanical axis shaft angle, plateau angle, and ankle tilt angle values were significantly higher in individuals aged 62–75 years. According to Kellgren–Lawrence staging, significant increases were observed in Q angle, hip–knee–ankle angle, femoral mechanical axis shaft angle, plateau angle, and ankle tilt angle in advanced-stage disease (Stage 4) (*p* < 0.05). Although mechanical lateral distal femoral angle, femoral Q angle, tibial Q angle, condylar plateau angle, and tibiotalar angle showed numerical differences across disease stages, these findings did not reach statistical significance. *Conclusions:* Comprehensive assessment of lower-extremity alignment may provide complementary information regarding biomechanical changes associated with knee osteoarthritis progression and could support individualized clinical follow-up and treatment planning.

## Simple Summary

Knee osteoarthritis (OA) is linked to changes in lower limb alignment that may affect disease severity and progression. In this study, radiographic measurements were used to analyze hip–knee–ankle alignment parameters across different age groups and disease stages. Notable differences were observed in several angular values, especially in older individuals and advanced OA stages. These results emphasize the clinical value of detailed alignment evaluation for better diagnosis and more personalized management strategies in knee osteoarthritis.

## 1. Introduction

Osteoarthritis (OA) is a disease characterized by structural changes in the joint cartilage of synovial joints [1,2,3]. During the progression of OA, degenerative changes occur in the joint cartilage, joint capsule, ligaments, periarticular muscles, synovial membrane, and subchondral bone [4,5,6,7]. The initial degenerative changes at the tissue level include cartilage fibrillation, followed by a decrease in proteoglycan and water content [8]. In OA, particularly, the loss of joint cartilage is associated with a decrease in water content, disruption in the synthesis of collagen and proteins necessary for cartilage repair, and weakening of the cartilage tissue [9]. In the final stages, the load-bearing capacity of the cartilage diminishes, leading to degeneration in the subchondral bone. This condition is often accompanied by osteophyte formation and cystic changes. Osteophytes, which develop at the joint margins in OA, result from the outward growth of bone. This bone growth at the joint edges restricts joint movement and may lead to joint fusion [10,11].

OA is classified based on the number of joints affected as monoarticular, oligoarticular, or polyarticular [12,13,14]. It is further categorized into primary and secondary OA based on its etiology. The cause of primary OA is unknown, while secondary OA is associated with metabolic factors (e.g., calcium crystal deposition disease, acromegaly), anatomical abnormalities (e.g., Legg-Perthes disease, epiphyseal dysplasias, congenital hip dislocation, lower extremity length discrepancies), traumatic events (e.g., intraarticular fractures, primary joint trauma, chronic injuries), and inflammatory conditions (e.g., inflammatory arthropathies, septic arthritis) [15,16,17,18].

For patients at high risk, lifestyle changes aimed at preventing or slowing the progression of knee OA are crucial [19,20]. Early identification of underlying alignment disorders allows for the implementation of joint-protective strategies [21]. Incorporating specific clinical parameters into patient assessments could enhance treatment outcomes. Therefore, studies focused on lower extremity alignment in patients with knee OA are essential, as they contribute valuable data to treatment strategies [10,12,13,15,22,23,24].

Factors affecting a joint can lead to changes in the vertical anatomical alignment of the lower extremity through compensatory mechanisms that impact the joints above and below [8,25,26]. It is important to analyze these changes, as they can significantly affect individuals’ quality of life by influencing the interconnected joints vertically [27,28]. The aim of this study is to evaluate the anatomical alignment profile of the hip, knee, and ankle as a whole in patients with knee OA at different stages. This study aims to comprehensively evaluate established anatomical and mechanical lower-extremity alignment parameters that may provide complementary information for the radiographic assessment and longitudinal follow-up of patients with knee osteoarthritis.

Although several lower-extremity alignment parameters have previously been investigated individually in patients with knee osteoarthritis, comprehensive studies integrating multiple anatomical and mechanical measurements across different Kellgren–Lawrence stages, age groups, and sexes within a single cohort remain limited. The present study aims to provide a holistic radiographic characterization of lower-extremity alignment patterns and to explore the interrelationships among these parameters throughout disease progression. Rather than introducing entirely novel parameters, our approach emphasizes the simultaneous and integrated evaluation of established measurements across age groups, sexes, and Kellgren–Lawrence stages, thereby providing a more comprehensive characterization of lower-extremity alignment patterns in knee osteoarthritis.

## 2. Materials and Methods

This retrospective cross-sectional study aimed to evaluate radiographic lower extremity alignment parameters in patients with knee osteoarthritis across different Kellgren–Lawrence stages, age groups, and sexes.

### 2.1. Radiography

Direct radiography is the most commonly used radiological method for diagnosing OA and planning treatment. In our study, patients were grouped according to their stages using the Kellgren-Lawrence (K-L) classification system, the most commonly used radiological grading system in knee OA. The angle and length values of the patients were examined in terms of stage, gender, and age groups.

### 2.2. Participants

The study included 200 patients diagnosed with knee osteoarthritis who were treated at the Department of Orthopedics and Traumatology, Ege University Faculty of Medicine.

Inclusion criteria were: (1) age between 40 and 75 years, (2) a radiographic diagnosis of knee osteoarthritis, and (3) availability of complete standing anteroposterior lower-extremity radiographs.

Exclusion criteria included a history of lower-extremity surgery, meniscal injury, ankle instability, bursitis, plantar fasciitis, congenital or acquired lower-limb deformities, neurological disorders affecting gait or posture, and any other orthopedic conditions that could influence lower-extremity alignment measurements.

Due to the retrospective nature of the study and the use of anonymized radiographic data, the requirement for informed consent was waived by the Institutional Ethics Committee (Decision No. 21-8T/12, 26 August 2021).

### 2.3. Data Collection Tools

X-ray images of patients with knee OA were obtained from the Hospital Information System of Ege University. All lower extremity radiographs were taken with the patient in a natural position, ensuring visibility of internal and external rotation in the standing position. Three X-ray images per patient, including the entire lower extremity, knee joint, and ankle joint in anteroposterior views, were analyzed. A total of 400 lower extremities and 1200 images were evaluated. The radiological images were transferred to an Apple MacBook Pro (13-inch, M1, 2020; Apple Inc., Cupertino, CA, USA) and measured in a standardized manner using the ImageJ software program (version 1.53k; National Institutes of Health, Bethesda, MD, USA) for distances and angles between the anatomical points identified in the study (OB). Two observers utilized the ImageJ software to assess lower extremity alignment. Each parameter was measured three times, and the average of these measurements was calculated.

### 2.4. Classification of Patients According to Kellgren-Lawrence Osteoarthritis Staging

**Figure 1:**
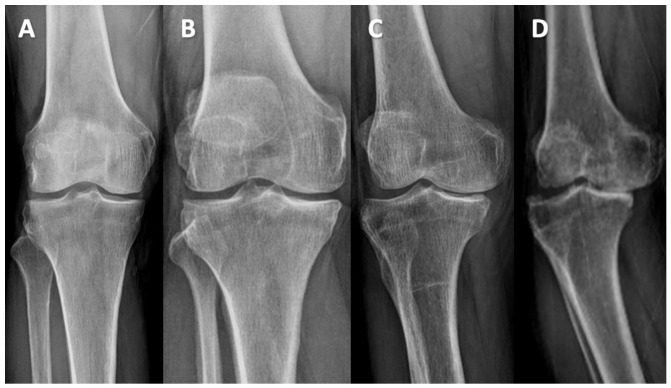
Classification of patients with knee osteoarthritis according to K-L stages. Stage 1 (**A**), Stage 2 (**B**), Stage 3 (**C**), and Stage 4 (**D**) patients’ anteroposterior knee joint X-ray images.

In the radiological examination, volunteer patients were classified into four stages according to the Kellgren-Lawrence classification (K-L staging), a widely used system in orthopedics (Figure 1) [29].

### 2.5. Identification of Anatomical Landmarks on Radiographs

The anatomical landmarks selected for radiographic evaluation comprised the anterior superior iliac spine, pubic symphysis, center of the femoral head, greater trochanter, medial femoral condyle, lateral femoral condyle, midpoint of the patella, medial tibial condyle, lateral tibial condyle, tibial tuberosity, trochlea tali, medial malleolus, and lateral malleolus. These reference points were identified on all radiographic images and subsequently used for morphometric analyses.

### 2.6. Image Processing and Measurement Procedure Using ImageJ Software

All radiographic images obtained from patients diagnosed with knee osteoarthritis were imported into the ImageJ software environment through the “Open” command available in the “File” menu. To establish measurement calibration, a 10-cm reference line corresponding to the scale marker visible on each radiograph was drawn, and the associated pixel value was recorded. Following calibration, the reference ruler was removed, and the pixel-to-distance conversion was defined using the “Set Scale” function located within the “Analyze” menu. Subsequently, angular and linear measurements were performed for each image. This procedure was conducted independently for all 200 radiographs, and the resulting measurements were systematically documented in Microsoft Excel for further analysis.

### 2.7. Radiographic Parameters Evaluated for Lower Extremity Alignment Analysis

**Figure 2:**
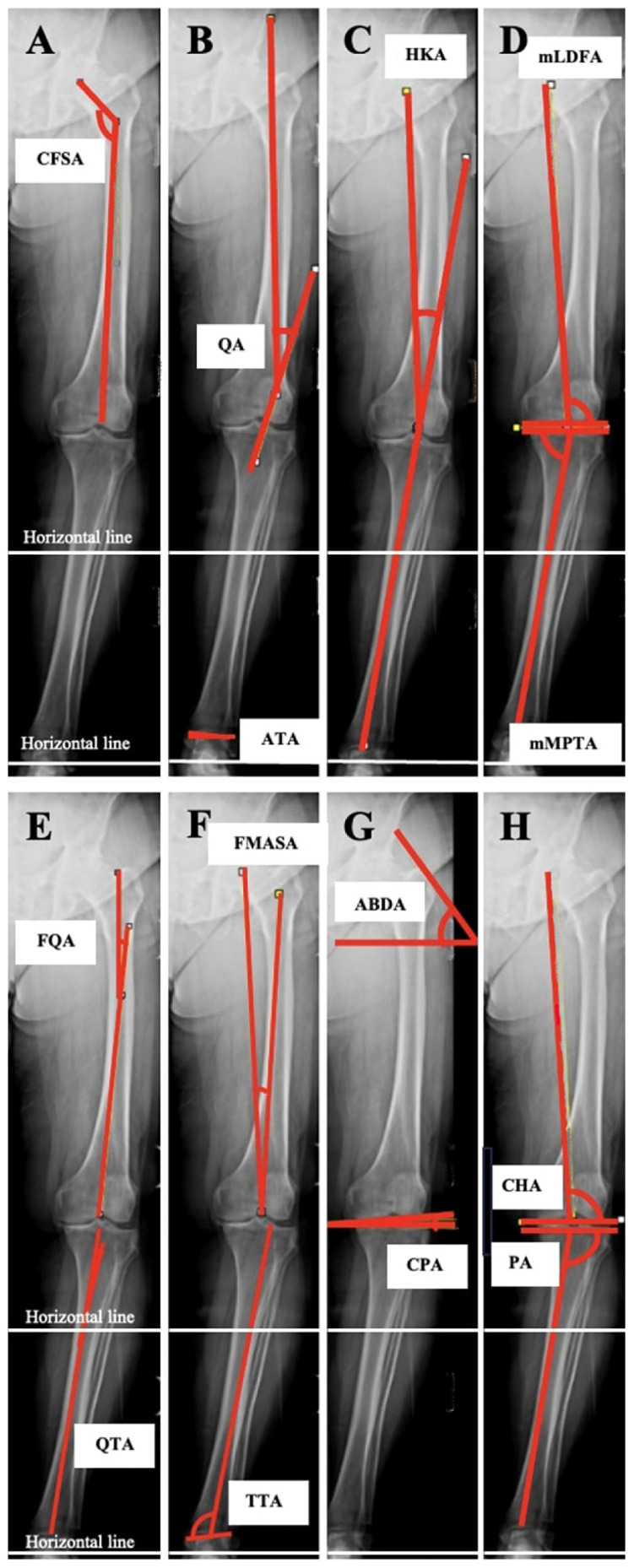
Definition of anatomical landmarks and parameters. (**A**): Collum femoris shaft angle (**CFSA**), (**B**): Q angle (**QA**), ankle tilt angle (**ATA**), (**C**): Hip, knee, ankle angle (**HKA**), (**D**): Mechanical lateral distal femoral angle (**mLDFA**), mechanical medial proximal tibial angle (**mMPTA**), (**E**): Femoral Q angle (**FQA**), tibial Q angle (**QTA**), (**F**): Femoral mechanical axis shaft angle (**FMASA**), tibiotalar angle (**TTA**), (**G**): Abductor angle (**ABDA**), condylar plateau angle (**CPA**), (**H**): Condylar hip angle (**CHA**), plateau angle (**PA**).

A total of 24 radiographic parameters were defined to assess lower-extremity alignment characteristics in patients with knee osteoarthritis. Except for hip center length, all measurements were obtained bilaterally from both lower extremities (Figure 2). Consequently, 47 individual measurements were recorded for each participant.

*Anatomical Femoral Length (AFL):* Anatomical femoral length was defined as the distance extending from the proximal end of the femur to the midpoint between the medial and lateral femoral condyles, representing the division point of the femoral anatomical axis. The knee joint center was determined as the midpoint located between the medial and lateral femoral condyles.

*Mechanical Femoral Length (MFL):* The distance from the center of the caput femoris to the midpoint between the condylus medialis femoris and condylus lateralis femoris.

*Anatomical Tibial Length (ATL):* The length of the line extending from the tibial plateau to the transmalleolar axis, dividing the long axis of the tibia into two.

*Mechanical Tibial Length (MTL):* The distance between the midpoint of the tibial plateau and the midpoint of the transmalleolar axis.

*Abductor Length (ABDL):* The length of the line perpendicular to the line drawn from the center of the caput femoris to the lateral side of the trochanter major.

*Abductor Angle (ABDA):* The angle between the line drawn from the lateral edge of the trochanter major and a horizontal line (Figure 2G).

*Length of the Hip Center (LHC):* LHC was defined as the perpendicular distance extending from the horizontal reference line passing through the center of the femoral head to the inferior margin of the ischium. The center of the hip joint was considered the midpoint of the femoral head.

*Lever Arm of Body Weight (LABW):* LABW was measured as the horizontal distance between the center of the femoral head and the vertical reference line passing through the pubic symphysis.

*Femoral Offset (FO):* FO was determined as the distance from the center of the femoral head to the superior border of the femoral shaft axis.

*Collum Femoris Length (CFL):* CFL was defined as the linear distance extending from the center of the femoral head to the point where the femoral neck axis intersects the femoral shaft axis.

*Collum Femoris Shaft Angle (CFSA):* CFSA was measured as the angle formed between the longitudinal axis of the femoral neck and the anatomical axis of the femoral shaft (Figure 2A).

*Q Angle (QA):* QA was defined as the angle created by the intersection of a line extending from the anterior superior iliac spine to the midpoint of the patella and a second line connecting the tibial tuberosity to the midpoint of the patella (Figure 2B).

*Hip–Knee–Ankle Angle (HKA):* HKA represented the angle formed between the mechanical axes of the femur and tibia (Figure 2C).

*Mechanical Lateral Distal Femoral Angle (mLDFA):* mLDFA was defined as the lateral angle between the distal femoral joint line, drawn tangentially to the medial and lateral femoral condyles, and the mechanical axis of the femur (Figure 2F).

*Mechanical Medial Proximal Tibial Angle (mMPTA):* mMPTA was measured as the medial angle between the proximal tibial joint line and the mechanical axis of the tibia (Figure 2D). Physiological values of both mLDFA and mMPTA generally range between 85° and 95°.

*Femoral Q Angle (FQA):* FQA was defined as the angle formed by the lines connecting the proximal and distal midcortical centers of the femur (Figure 2E).

*Tibial Q Angle (TQA):* TQA was measured as the angle between the lines connecting the proximal and distal midcortical centers of the tibia (Figure 2E). A lateral curvature exceeding 2° was classified as genu varum, whereas a medial curvature greater than 2° was classified as genu valgum.

*Trochlear Width (TW):* TW was defined as the distance between points h and i, identified by the intersections of predefined lines on the medial and lateral margins of the trochlea. The first and second lines intersected parallel reference lines, whereas the third line connected the inferior borders of the medial and lateral femoral condyles.

*Femoral Mechanical Axis Shaft Angle (FMASA):* FMASA was measured as the angle between the mechanical axis of the femur and the anatomical axis of the femoral shaft (Figure 2F).

*Condylar Plateau Angle (CPA):* CPA was defined as the angle formed between a horizontal line tangent to the superior articular surface of the tibia and a line passing through the inferior borders of the medial and lateral femoral condyles (Figure 2G).

*Plateau Angle (PA):* PA was measured as the lateral angle between the line contacting the superior articular surface of the tibia and the mechanical axis of the tibia (Figure 2H).

*Condylar Hip Angle (CHA):* CHA was defined as the lateral angle between the transcondylar axis of the femur and the mechanical axis of the femur (Figure 2H).

*Ankle Tilt Angle (ATA):* ATA was measured as the angle between the line extending from the trochlea tali and the horizontal reference line (Figure 2B). The center of the ankle joint was defined as the midpoint of the trochlea tali.

*Tibiotalar Angle (TTA):* TTA was defined as the angle between the line extending from the trochlea tali and the anatomical axis of the tibia (Figure 2F).

### 2.8. Radiographic Acquisition Protocol

All long-leg radiographs were obtained in a standardized standing weight-bearing position. Participants stood in a double-leg stance with both knees fully extended, the patellae oriented anteriorly, and the feet maintained in a neutral position to minimize rotational variations. A fixed source-to-image distance was used according to institutional radiographic protocols. Calibration of all measurements was performed using the digital tools available in ImageJ software. These procedures were implemented to reduce positioning-related variability in lower-extremity alignment parameters, including HKA, mLDFA, and mMPTA.

### 2.9. Data Evaluation

Statistical analyses were conducted using IBM SPSS Version 25.0. The dependent sample *t*-test was applied to compare lower extremity measurement values between the right and left sides. One-way ANOVA (Analysis of Variance) was used to evaluate lower extremity length and angle values according to age groups and Kellgren-Lawrence (K-L) stages (Table 1 and Table 2). Pearson Correlation Analysis was utilized to determine the direction and strength of the relationship between lower extremity measurements and patients’ age and Kellgren-Lawrence stages. Additionally, Pearson Correlation Analysis was applied to examine the relationship between lower extremity angle measurements and the hip-knee-ankle angle. To improve the interpretability and precision of the reported associations, 95% confidence intervals were calculated and presented alongside correlation coefficients and *p*-values.

Both lower extremities of each participant were included in the analyses, and the lower limb was considered the unit of analysis. Since the primary aim of the study was to comprehensively characterize radiographic alignment parameters, measurements from both sides were evaluated. The potential influence of within-subject correlations between the right and left lower extremities is acknowledged and should be considered when interpreting the findings.

## 3. Results

**Figure 3:**
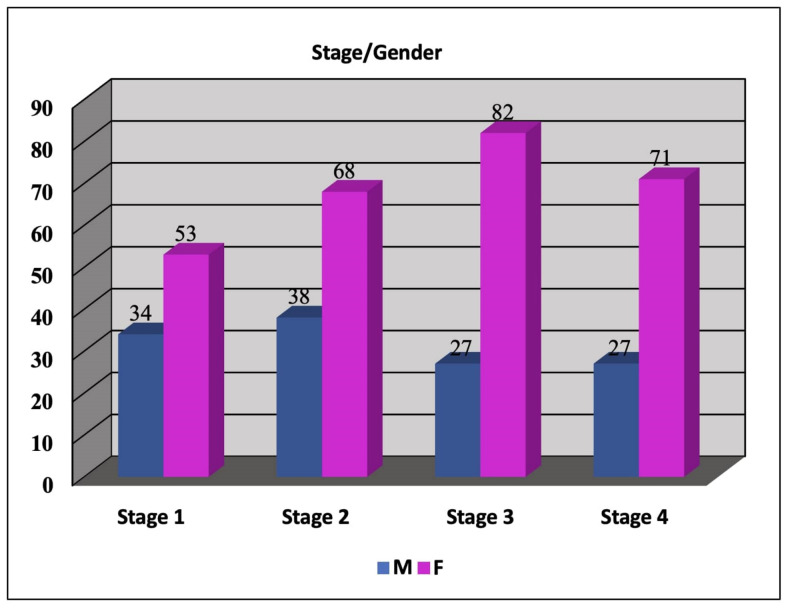
Distribution of male and female patients across Kellgren–Lawrence stages of knee osteoarthritis.

In patients with knee OA, the female-to-male ratio was 2.17:1 (137 females/63 males). Of the lower extremities evaluated, 21.75% were classified as Stage 1 (87 lower extremities, 53 females, 34 males), 26.5% as Stage 2 (106 lower extremities, 68 females, 38 males), 27.25% as Stage 3 (109 lower extremities, 82 females, 27 males), and 24.5% as Stage 4 (98 lower extremities, 71 females, 27 males) (Figure 3).

A dependent-sample T-test was conducted to compare the measurement values of the lower extremities on the right and left sides. Anatomical femoral length, mechanical femoral length, anatomical tibial length, mechanical tibial length, collum femoris length, trochlear width, hip-knee-ankle angle, femoral mechanical axis shaft angle, condylar plateau angle, plateau angle, and condylar hip angle were significantly lower on the right side; abductor length, abductor angle, mechanical medial proximal tibial angle, and femoral Q angle were found to be lower on the left side (*p* < 0.05).

Men had higher values for anatomical femoral length, mechanical femoral length, anatomical tibial length, mechanical tibial length, abductor length, length of the hip center, femoral deviation, collum femoris length, trochlear width, right plateau angle, and left tibiotalar angle compared to women. In contrast, women had higher values for left body weight lever arm, left Q angle, hip-knee-ankle angle, and condylar plateau angle compared to men (*p* < 0.05).

One-way ANOVA was also applied to evaluate lower extremity length and angle values of patients with knee OA according to Kellgren-Lawrence stages (Table 2). Anatomical femoral length, mechanical femoral length, and trochlear width values were found to be lower in patients with advanced-stage disease compared to those with early-stage disease (*p* < 0.05).

These findings demonstrate differences in selected anatomical and mechanical measurements across Kellgren–Lawrence stages; however, due to the cross-sectional design, no causal relationship regarding OA progression can be inferred.

Pearson correlation analysis was performed to evaluate both the magnitude and direction of the associations between lower-extremity angular parameters and hip–knee–ankle (HKA) angle measurements.

Weak negative correlations were found between anatomical femoral length, mechanical femoral length, anatomical tibial length, mechanical tibial length, abductor length, right body weight lever arm, femoral offset, collum femoris length, and the angle values of the hip-knee-ankle on both sides (r = −0.309 to −0.132, 95% CI: −0.47 to −0.18, *p* < 0.05).

Moderate negative correlations were found between right trochlear width and the angle values of the right hip-knee-ankle (r = −0.511, 95% CI: −0.64 to −0.35, *p* < 0.05) and between left trochlear width and the angle values of the left hip-knee-ankle (r = −0.497, 95% CI: −0.64 to −0.31, *p* < 0.05). Additionally, negligible correlations were observed between right and left abductor length and the hip-knee-ankle angle values (right r = −0.039, 95% CI: −0.16 to 0.22, *p* > 0.05; left r = −0.046, 95% CI: −0.149 to 0.232, *p* > 0.05), as well as between the length of the hip center, right body weight lever arm, and the right hip-knee-ankle angle values (r = −0.03 to −0.16, 95% CI: −0.22 to 0.37, *p* > 0.05).

Strong positive correlations were observed between the right Q angle and the right HKA angle (r = 0.662, 95% CI: 0.48 to 0.8, *p* < 0.05) and between the left Q angle and the left HKA angle (r = 610, 95% CI: 0.39 to 0.82, *p* < 0.05). Moderate positive correlations were observed between the right Q angle and the left HKA angle (r = 0.545, 95% CI: 0.36 to 0.68, *p* < 0.05), as well as between the left Q angle and the right HKA angle (r = 0.537, 95% CI: 0.36 to 0.68, *p* < 0.05). Furthermore, a strong positive correlation was observed between right and left HKA angle measurements (r = 0.771, 95% CI: 0.61 to 0.88, *p* < 0.05). Strong positive correlations were also detected between the left mechanical lateral distal femoral angle (mLDFA) and the left HKA angle (r = 0.697, 95% CI: 0.52 to 0.80, *p* < 0.05) and between the right mLDFA and the right HKA angle (r = 0.714, 95% CI: 0.53 to 0.82, *p* < 0.05).

Conversely, strong inverse correlations were observed between the mechanical right and left medial proximal tibial angle (mMPTA) and HKA measurements (right r = −0.634, 95% CI: −0.77 to −0.4, *p* < 0.05; left r = −0.695, 95% CI: −0.81 to −0.52, *p* < 0.05), while the association between the right and left abductor angle and HKA values was characterized as weakly (right r = −0.016, 95% CI: −0.21 to 0.15, *p* > 0.05; left r = 0.057, 95% CI: −0.16 to 0.25, *p* > 0.05).

Moderate positive correlations were additionally observed between the right and left femoral Q angle (FQA) and HKA angle (right r = 0.4, 95% CI: 0.1 to 0.61, *p* < 0.05; left r = 0.436, 95% CI: 0.21 to 0.62, *p* < 0.05), right and left plateau angle (PA) and HKA angle (r = 0.575, 95% CI: 0.4 to 0.7, *p* < 0.05; r = 0.485, 95% CI: 0.31 to 0.66, *p* < 0.05), right and left ankle tilt angle (ATA) and HKA measurements (right r = 0.425, 95% CI: 0.12 to 0.69, *p* < 0.05; left r = 0.439, 95% CI: 0.19 to 0.61, *p* < 0.05).

Weak positive correlations were observed between HKA measurements and the fol-lowing parameters: tibial Q angle (TQA), collum femoris shaft angle (CFSA), left tibial Q angle, femoral mechanical axis shaft angle (FMASA), condylar plateau angle (CPA), condylar hip angle (CHA), and tibiotalar angle (TTA); however, these associations did not reach statistical significance (r = 0.2 to 0.39, 95% CI: −0.14 to 4.6, *p* > 0.05).

In patients with advanced-stage knee osteoarthritis, higher HKA values were observed in patients with advanced varus deformity. In contrast, mMPTA values tended to be lower than those observed in individuals with early-stage disease; however, this difference did not reach statistical significance (*p* = 0.073).

## 4. Discussion

Knee osteoarthritis (OA) is a prevalent degenerative musculoskeletal disorder, particularly among older adults, and radiographic assessment remains a fundamental tool for evaluating disease severity. The Kellgren–Lawrence classification has long been used to characterize OA progression, primarily based on joint-space narrowing, osteophyte formation, and subchondral sclerosis [1,28,30,31,32]. However, structural alterations associated with OA may extend beyond the knee joint and involve the overall alignment of the lower extremity.

The present study showed that several lower-extremity alignment parameters varied across Kellgren–Lawrence stages and age groups. Individuals with advanced-stage OA generally exhibited greater deviations from physiological alignment than those with early-stage disease. These findings are consistent with previous studies reporting increased varus alignment and altered hip–knee–ankle relationships in advanced OA [30,33,34]. Nevertheless, because of the cross-sectional nature of our study, these findings should be interpreted as descriptive associations rather than evidence of causal mechanisms or disease progression.

Age-related differences were also observed in several measurements. Earlier reports have shown that advanced OA stages are more common among older individuals and are accompanied by alterations in lower-limb alignment [13,17,18,28,30]. Our findings further indicate that age and disease severity may jointly influence lower-extremity alignment patterns. However, age itself should not be regarded as an independent causal factor based on the present data.

Sex-related differences in certain linear measurements, including hip center and collum femoris lengths, were identified. Similar findings have been described previously, with anatomical dimensions differing according to sex and age [12,16,20,32,35,36,37]. Importantly, anthropometric variables such as body height, weight, and body mass index were unavailable in our retrospective dataset. Therefore, these differences should be interpreted cautiously, as they may partially reflect variations in body size rather than osteoarthritis-specific anatomical changes.

Previous studies have emphasized the importance of the hip–knee–ankle axis in maintaining functional lower-extremity alignment [30]. In our cohort, changes in hip, knee, and ankle alignment appeared to occur concurrently across disease stages, suggesting that OA affects interconnected anatomical regions rather than isolated structures. Parameters with *p*-values greater than 0.05 were not considered statistically significant and were interpreted cautiously as descriptive observations.

The clinical relevance of our results lies in highlighting the integrated nature of lower-extremity alignment in patients with knee OA. Comprehensive radiographic assessment may provide additional information regarding alignment patterns and could complement conventional clinical and radiological evaluations. However, these measurements should be considered supportive tools and not replacements for established diagnostic or treatment-planning approaches.

Several limitations of this study should be acknowledged. First, this was a single-center, retrospective, cross-sectional investigation; therefore, causality and true osteoarthritis progression could not be directly assessed. Moreover, the absence of a healthy control group precludes determining whether the observed alignment characteristics are specific to knee osteoarthritis or reflect age-related anatomical variation.

Second, important clinical and anthropometric variables, including body height, weight, body mass index (BMI), pain severity, functional scores, compartment involvement, and affected side, were unavailable in the retrospective database. The absence of these variables may have influenced linear radiographic measurements and contributed to the observed age- and sex-related differences. Future studies incorporating these parameters into multivariable models are warranted to better distinguish body-size effects from osteoarthritis-related anatomical changes. Furthermore, the lack of information regarding the predominantly affected compartment, affected side, pain severity, and functional status limits the clinical interpretation of the observed alignment patterns and prevents determination of whether these findings are specific to osteoarthritis or reflect broader age-related anatomical variation.

Third, both lower extremities from the same individuals were included in the analyses, which may have partially violated the assumption of independence between observations. Future investigations employing mixed-effects models or generalized estimating equations may provide more robust estimates for paired measurements.

Additional exploratory sensitivity analyses, including paired comparisons and profile plots with 95% confidence intervals, showed comparable right- and left-sided trajectories across Kellgren–Lawrence stages for representative alignment parameters. Although some side-to-side differences were observed, the principal findings and overall interpretation remained unchanged, suggesting that the inclusion of both extremities did not materially influence the study conclusions.

Fourth, formal inter-observer and intra-observer reliability analyses, such as intraclass correlation coefficients (ICCs) or Bland–Altman assessments, were not performed. Although all measurements were independently repeated by experienced observers, reproducibility metrics should be incorporated in future studies to further validate measurement consistency.

Finally, the statistical analyses were primarily based on group comparisons and correlation analyses. Multivariable approaches accounting for potential confounding factors, including age, sex, anthropometric characteristics, and disease severity, may provide a more comprehensive understanding of independent associations and should be considered in future investigations.

In conclusion, our findings suggest that lower-extremity alignment characteristics vary according to age and Kellgren–Lawrence stage in patients with knee OA. Greater deviations from physiological alignment patterns were observed in advanced disease stages. These findings should be interpreted cautiously given the retrospective cross-sectional design of the study. Prospective, longitudinal, and multicenter studies incorporating anthropometric variables and comprehensive biomechanical assessments are needed to clarify the clinical significance of these radiographic findings.

## 5. Conclusions

In conclusion, lower-extremity alignment characteristics varied according to age and Kellgren–Lawrence stage in patients with knee osteoarthritis. Given the absence of anthropometric, clinical, and functional variables as well as a healthy control group, these findings should be interpreted as descriptive associations rather than definitive indicators of disease-specific mechanisms or clinical outcomes. These findings should be interpreted within the context of a cross-sectional design and do not imply causality. Prospective multicenter longitudinal studies incorporating clinical and anthropometric variables are needed to clarify the clinical relevance of these findings.

## Figures and Tables

**Table 1:** Comparison of lower limb angle values between the younger and older patients.

Parameters (Right/Left)	40–50 Years (Mean ± SD)	51–61 Years (Mean ± SD)	62–75 Years (Mean ± SD)	*p* Value
**Q angle**	11.29 ± 2.15	11.56 ± 2.72	13.62 ± 3.56	<0.001 *
	11.81 ± 1.94	12.22 ± 2.77	13.37 ± 3.8	0.001 *
**Abductor angle**	70.93 ± 2.92	70.62 ± 3.2	70.55 ± 4.36	0.873
	69.66 ± 2.74	68.71 ± 3.23	69.12 ± 4.36	0.419
**Hip, knee, ankle angle**	7.66 ± 3.68	9.11 ± 3.99	11.46 ± 5.39	<0.001 *
	8.43 ± 3	9.69 ± 4.55	11.66 ± 5.96	0.002 *
**Collum femoris shaft angle**	134.04 ± 5.56	132.81 ± 5.37	133.52 ± 6.27	0.523
	133.11 ± 4.78	132.16 ± 5.2	133.35 ± 5.7	0.344
**Mechanical lateral distal femoral angle**	90.37 ± 3.12	91.48 ± 3.13	92.31 ± 4.07	0.022 *
	90.31 ± 3.38	91.11 ± 2.93	91.64 ± 3.86	0.148
**Mechanical medial proximal tibial angle**	85.59 ± 3.64	84.96 ± 3.34	83.89 ± 3.57	0.029 *
	85.39 ± 2.69	84.42 ± 2.66	83.11 ± 3.47	<0.001 *
**Femoral Q angle**	2.21 ± 1.24	2.94 ± 2.51	3.8 ± 3.29	0.009 *
	2.16 ± 1.31	2.12 ± 1.63	2.75 ± 2.41	0.087
**Tibial Q angle**	2.26 ± 1.7	2.73 ± 1.99	3.38 ± 2.5	0.023 *
	2.24 ± 1.77	2.5 ± 1.86	2.91 ± 2.16	0.187
**Femoral mechanical axis shaft angle**	5.53 ± 0.85	5.83 ± 0.67	5.79 ± 0.93	0.156
	5.76 ± 0.76	6.01 ± 0.69	5.93 ± 0.71	0.226
**Condylar plateau angle**	2.93 ± 0.67	2.92 ± 0.67	3.16 ± 0.86	0.098
	3.19 ± 0.67	3.2 ± 0.8	3.37 ± 0.87	0.309
**Plateau angle**	90.89 ± 2.96	90.82 ± 2.11	91.69 ± 2.29	0.048 *
	91.4 ± 2.17	91.66 ± 2.07	92.35 ± 2.11	0.034 *
**Condylar hip angle**	85.22 ± 2.93	85.44 ± 2.88	84.94 ± 2.79	0.533
	86.46 ± 2.28	85.98 ± 2.48	85.3 ± 3.01	0.068
**Ankle tilt angle**	3.71 ± 2.15	4.12 ± 2.92	4.69 ± 3.44	0.221
	3.83 ± 2.54	4.04 ± 3.16	4.94 ± 3.39	0.103
**Tibiotalar angle**	94.12 ± 4.61	94.61 ± 3.95	95.78 ± 4.75	0.139
	94.54 ± 4.53	94.24 ± 3.94	95.63 ± 5.12	0.104

Values are presented as mean ± SD (standard deviation). For each parameter, the first row corresponds to right-sided measurements and the second row corresponds to left-sided measurements. Statistically significant differences are indicated by * (*p* < 0.05).

**Table 2:** Differences in lower limb angle values in patients with knee osteoarthritis according to K-L stages.

Parameters (Right/Left)	Stage 1 (Mean ± SD)	Stage 2 (Mean ± SD)	Stage 3 (Mean ± SD)	Stage 4 (Mean ± SD)	*p* Value
**Q angle**	11.18 ± 1.84	11.08 ± 1.99	12.77 ± 2.82	15.33 ± 3.44	<0.001 *
	10.78 ± 1.26	11.39 ± 2.25	12.69 ± 3.11	14.4 ± 4.22	
**Abductor angle**	70.5 ± 3.75	70.35 ± 3.98	71.17 ± 2.91	70.66 ± 3.84	0.172
	69.12 ± 3.39	69.37 ± 3.77	68.91 ± 3.54	68.85 ± 4.02	
**Hip, knee, ankle angle**	6.51 ± 2.11	7.39 ± 2.02	10.37 ± 3.61	14.57 ± 5.39	<0.001 *
	7.41 ± 1.94	7.93 ± 2.42	10.49 ± 4.29	15.2 ± 6.5	
**Collum femoris shaft angle**	132.7 ± 4.96	132.57 ± 5.39	133.11 ± 5.41	134.85 ± 6.86	0.053
	132.42 ± 5.36	133.42 ± 5.39	131.95 ± 4.85	133.79 ± 5.83	
**Mechanical lateral distal femoral angle**	89.63 ± 2.6	90.45 ± 2.13	91.78 ± 2.92	94.33 ± 4.41	0.064
	90.12 ± 2.83	90.1 ± 2.46	91.2 ± 2.81	93.32 ± 4.57	
**Mechanical medial proximal tibial angle**	85.87 ± 2.55	85.77 ± 2.31	84.86 ± 2.66	82.23 ± 4.69	0.073
	85.33 ± 2.59	85.55 ± 2.37	83.67 ± 2.8	81.82 ± 3.34	
**Femoral Q angle**	2.15 ± 1.41	2.32 ± 1.81	3.37 ± 2.73	4.68 ± 3.64	0.577
	2.06 ± 1.21	1.89 ± 1.57	2.1 ± 1.6	3.62 ± 2.76	
**Tibial Q angle**	2.61 ± 1.99	2.23 ± 1.56	2.94 ± 2.08	3.85 ± 2.71	0.563
	2.48 ± 1.76	2.26 ± 1.66	2.83 ± 2.18	2.81 ± 2.18	
**Femoral mechanical axis shaft angle**	5.66 ± 0.6	5.83 ± 0.82	5.75 ± 0.84	5.75 ± 0.95	0.034 *
	5.82 ± 0.62	5.95 ± 0.75	6 ± 0.7	5.92 ± 0.77	
**Condylar plateau angle**	2.94 ± 0.63	3.04 ± 0.8	2.99 ± 0.72	3.08 ± 0.84	0.244
	3.21 ± 0.72	3.28 ± 0.83	3.13 ± 0.75	3.49 ± 0.93	
**Plateau angle**	90.58 ± 1.61	90.43 ± 1.82	91.45 ± 1.99	92.27 ± 3.22	0.043
	91.04 ± 1.82	91.29 ± 1.65	91.88 ± 1.95	93.39 ± 2.37	
**Condylar hip angle**	85.23 ± 2.56	85.56 ± 3.33	84.49 ± 2.39	85.39 ± 2.82	0.256
	86.21 ± 2.05	85.81 ± 2.89	85.38 ± 2.88	85.94 ± 2.81	
**Ankle tilt angle**	3.03 ± 1.9	3.58 ± 2.63	4.29 ± 2.65	6.02 ± 3.7	<0.001 *
	3.2 ± 2.19	3.7 ± 2.82	4.78 ± 3.41	5.64 ± 3.52	
**Tibiotalar angle**	93.74 ± 3.75	94.09 ± 4.22	95.41 ± 4.67	96.63 ± 4.54	0.124
	94.15 ± 4.22	94.25 ± 4.24	94.79 ± 4.63	96.29 ± 5.02	

Values are presented as mean ± standard deviation. For each parameter, the first row corresponds to right-sided measurements and the second row corresponds to left-sided measurements. Statistically significant differences are indicated by * (*p* < 0.05).

## Data Availability

The datasets analyzed during the current study are not publicly available due to ethical and institutional restrictions.

## Abbreviations

The following abbreviations are used in this manuscript:

OA	Osteoarthritis
K-L	Kellgren–Lawrence
AFL	Anatomical Femoral Length
MFL	Mechanical Femoral Length
ATL	Anatomical Tibial Length
MTL	Mechanical Tibial Length
ABDL	Abductor Length
ABDA	Abductor Angle
LHC	Length of the Hip Center
LABW	Lever Arm of Body Weight
FO	Femoral Offset
CFL	Collum Femoris Length
CFSA	Collum Femoris Shaft Angle
QA	Q Angle
HKA	Hip, Knee, Ankle Angle
mLDFA	Mechanical Lateral Distal Femoral Angle
mMPTA	Mechanical Medial Proximal Tibial Angle
FQA	Femoral Q Angle
QTA	Tibial Q Angle
TW	Trochlear Width
FMASA	Femoral Mechanical Axis Shaft Angle
CPA	Condylar Plateau Angle
PA	Plateau Angle
CHA	Condylar Hip Angle
ATA	Ankle Tilt Angle
TTA	Tibiotalar Angle
